# Cleft palate children: performance in auditory processing tests

**DOI:** 10.1016/S1808-8694(15)30780-1

**Published:** 2015-10-19

**Authors:** Mirela Boscariol, Karina Delgado André, Mariza Ribeiro Feniman

**Affiliations:** 1 Graduate Student. MSc–FCM Unicamp; 2 Specializing in Audiology, clinical speech and hearing therapist; 3 Associate Professor–Department of Speech and Hearing Therapy–Faculdade de Odontologia de Bauru, Universidade de São Paulo, USP-Bauru. University of São Paulo–Bauru Dentistry School–FOB USP Bauru Department of Speech and Hearing Therapy. Hospital de Reabilitação de Anomalias Craniofaciaias–HRAC

**Keywords:** hearing, children, cleft palate, auditory perception

## Abstract

Many children with auditory processing disorders have a high prevalence of otitis media, a middle ear alterations greatly prevalent in children with palatine and lip clefts.

**Aim:**

to check the performance of children with palate cleft alone (PC) in auditory processing tests. Prospective study.

**Materials and Methods:**

twenty children (7 to 11 years) with CP were submitted to sound location tests (SL), memory for verbal sounds (MSSV) and non verbal sounds in sequence (MSSNV), Revised auditory fusion (AFT-R), Pediatric test of speech intelligibility/synthetic sentences (PSI/SSI), alternate disyllables (SSW) and digit dichotic (DD). The children performances in the tests were classified in bad and good.

**Results:**

there was no statistically significant difference between genders and ears. The average values obtained were 2.16, 2.42, 4.37, 60.50ms; 40.71 to 67.33%; 96.25 to 99.38%; 73.55 to 73.88% and 58.38 to 65.47% respectively for the MSSNV, MSSV, LS, AFT-R, PSI/SSI tests with ipsilateral (PSI/SSIMCI) and contralateral (PSI/SSI/MCC) competitive message, DD and SSW tests.

**Conclusion:**

a high percentage of children showed worse results in the AFT-R, DD, SSW tests and in the PSI/SSIMCI tests. The best performances happened in the sound location tests, verbal and non-verbal sounds for sequential memory and for PSI/SSIMCC tests.

## INTRODUCTION

It is in the first years of life that auditory processing happens from experiencing worldly sounds. Thus, hearing loss and otitis media in this period, may be risk indicators for poor development, as well as for language, speech and learning disabilities, since this is a critical time–when children are learning to hear.^1^

Auditory processing is the term used to describe what happens when the brain recognizes and interprets the sounds around a person^2^; and auditory processing disorder refers to a difficulty in the perceptual processing of information in the Central Nervous System, shown by a low performance in one or more hearing skills, such as location, lateralization, auditory discrimination, auditory pattern recognition, temporal processing deficits and others.^3^

Thus, auditory processing and auditory processing disorders have been broadly studied in a variety of clinical studies^4^, ^5^, ^6^, ^7^, ^8^; however, very few reports are found in relation to central auditory function in the population with labio-palatine fissure. It is the large occurrence of otologic problems and the consequent peripheral hearing loss that have been stressed in those people with this congenital malformation.

Among otologic problems, otitis media with effusion (OME) has been reported as being almost universal in children with labio-palatine fissure.^9^^,^^10^ the main reason for this seems to be a failure in the Eustachian Tube opening, consequent to an abnormal insertion of the soft palate elevator and tensor muscles, causing Eustachian Tube obstruction and negative pressure in the middle ear^11^; Eustachian tube incapacity in balancing positive and negative pressures, because of its reduced elasticity and consequent functional obstruction^12^; abnormality in tube compliance13; tube patency–the property of the tube in opening up more than what is normal, allowing secretions to pass from the nasopharynx to the tympanic cavity^14^; and facial skeleton abnormalities^15^ have been suggested as contributing factors. Thus, a high occurrence of OME and hearing loss have been found in these children with labio-palatine fissure, even after palate repair, because despite soft palate tensor muscle improvement after this surgical procedure, it is hardly normalized, and the patient may still have constant otitis episodes^16^, ^17^, ^18^, ^19^, ^20^.

Otitis media with effusion produces mild to moderate and intermittent hearing loss, which impairs central auditory function. Central auditory alterations brought about by OME seem to add to the social and academic difficulties of these children.^21^

Then, the characterization of speech and hearing signs and symptoms, the search for an etiologic diagnosis and the finding of clinical entities that affect an individual with labio-palatine fissure have been the concern of health care professionals. Therefore, knowing that palatine fissure is a relevant craniofacial anomaly in our settings, and that according to the Joint Committee on Infant Hearing^22^ craniofacial anomalies represent a risk indicator for poor hearing, although not restricted to them, speech and hearing therapists, as members of a multidisciplinary team, besides understanding the determining causes of otologic complications present, must be able to assess the entire cochleovestibular system, and not only its peripheral segment, thus contributing to pursue proper approaches for prevention and treatment^23^.

## OBJECTIVE

To study the performance of children with palate fissure alone in special behavioral auditory processing tests.

## MATERIALS AND METHODS

20 children from both genders participated in this study, they were in the age range of 7 to 11 years (mean age of 9 years and 4 months), diagnosed as having palate fissure alone, chosen randomly in a hospital specialized in the care of such type of congenital malformation.

All the children had normal middle ear function and peripheral hearing, checked by means of conventional audiologic evaluation (audiometry and impedance testing); None of them had hearing complaints and/or upper airway disorders at the time of the tests, nor had they complaints of lack of attention, or any other difficulty to understand the tests.

Table 1 shows the distribution of patients according to age and gender.

**Table 1:** Children distribution according to age and gender.

Gender			Age (years)			
	7	8	9	10	11	Total
M	0	1	2	5	1	9
F	1	0	5	3	2	11
Total	1	1	7	8	3	20

Legend: M = male

F = female

This study was approved by the Ethics in Research Committee (protocol #: 876/2003UEPCEP) and the parents/guardians of the children who participated agreed to their participation and, after reading it, they signed the informed consent form. This study was carried out in the year of 2004.

The evaluation process was based on the application of special auditory processing behavioral tests, which were:

Diotic tests: Sound location test (LS)24, Verbal sequential memory test (MSSV)24, non-verbal sequential memory test(MSSNV) ^24^, and Auditory Fusion Test–Revised (AFT-R)^25^

Monotic tests: Pediatric Test of Speech Intelligibility with Ipsilateral Competitive Message (PSI/MCI)^26^ and the Synthetic Sentences Test with Ipsilateral Competitive

Message (SSI/MCI);^27^

Dichotic tests: Pediatric Test of Speech Intelligibility with Contralateral Competitive Message (PSI/MCC)^26^, Synthetic Sentences Test with Contralateral Competitive Message (SSI/MCC)^27^, Alternate Disyllable Test (SSW)^28^, and the Dichotic Digits Test (DD)^29^.

All the tests were performed in a sound-treated booth. For the monotic and dichotic tests we used the SD 50 two-channel audiometer hooked to a CD player.

The sound location test (LS) assesses the sound location skill^24^ and aims at providing information about the auditory physiological mechanism of sound source direct discrimination^30^. With the help of a rattle, five locations on the child's head were studied, with the eyes closed: to the right, to the left, in the front, behind, and above. The child should indicate where the sound was coming from. For such procedure, it is expected that the child gets at least four of the five directions correct^24^.

In the verbal sequential memory test (MSSV) the child is presented orally with three different sequences of syllables (PA, TA, CA), which the child should repeat in the exact sequence the sounds were presented. Children below 9 years of age have to get at least two of the three sequences correct. For children 9 years or older, it is expected that they are correct in all three different sequences presented^31^.

In the non-verbal sequential memory test (MSSNV) sound instruments were used (rattle, coconut, agogo and bells), sounded in three different sequences. The child was then asked to point to the instruments in the order they were used. It is expected that the child gets right at least two sequences of four sounds in three attempts.

The MSSV and MSSNV tests aimed at providing information about the physiological auditory mechanisms of sound discrimination in sequence^30^, aiming at assessing simple temporal ordering auditory skills.

LS, MSSV and MSSNV tests were shown to the children before being performed.

The AFT-R test is designed to measure temporal resolution, by determining the auditory fusion threshold, measured in milliseconds (ms). The children is presented with a series of pure tone pairs, separated by silence intervals from zero to 300ms, which he/she should answer if one or two tones were heard. For 7 year olds, it is expected to have fusion thresholds of 9ms (SD=4) and 8 ms (SD=3) for children from 8 years of age to 50 year old adults. It is employed a sound intensity of 50 dBSL, considering the air threshold of the frequencies assessed between 250 to 4,000 Hz.

The Pediatric Test of Speech Intelligibility^26^ and the Synthetic Sentences Test^27^ are phrases recognition tests by means of identifying figures (PSI) and written sentences (SSI), in the presence of ipsilateral competitive message (monotic hearing) and contralateral (dichotic hearing). The competitive message is a story. First the children are presented the figure recognition in PSI and the SSI sentences, from then on, the child is instructed to pay attention and point the figures or sentences corresponding to the phrase heard, disregarding the competitive message. The speech signal presentation intensity is 40dBSL, considering the mean value of tonal air thresholds in the frequencies of 500, 1,000 and 2,000Hz. We used the message/competition ratio of zero, −10 and −15, for the monotic hearing and zero and −40 for the dichotic, for both ears. It is expected that the child presents a percentage higher than or equal to 80, 70 and 60, respectively in the monotic hearing and higher than or equal to 90 and 100, respectively in the dichotic hearing.

Both the PSI and the SSI tests, assess the skill of background figure for verbal sounds30 and aim at providing information about the physiological auditory mechanism of verbal sound recognition in monotic and dichotic hearing.^30^

The dichotic digits test (DD)^29^ is based on the presentation of a list of 40 pairs of disyllable digits in Brazilian Portuguese (four, five, seven, eight and nine), in which four different digits are presented simultaneously, two in each ear, characterizing a dichotic task. We used the recorded version in the binaural integration stage. It is deployed at an intensity level of 50 dBSL, having as reference the mean values of the tonal air conduction thresholds in the frequencies of 500, 1,000 and 2,000Hz. The child is instructed to repeat all the digits heard in both ears. An error was considered when one digit was omitted or replaced. As normal values we considered 95% of correct answers or more in each ear for those aged 9 years or older and of 85% on the right and 82% on the left for those aged 7 and 8 years.

The alternate disyllable test–SSW^28^ is based on the presentation of 40 items, and each item formed by four paroxytone disyllables, making up a total of 160 words. In each item, two words were presented to each ear, and there was an overlapping between the second syllable of the second word and the first syllable of the third word, which were sent at the same time to opposite ears. Thus noticing for each item the DNC situation (word presented to the right ear without competitive message), DC (word presented to the right ear with concurrent competition to the left ear), EC (word presented to the left ear with concurrent competition to the right ear) and ENC (Word presented to the left ear without competitive message to the contralateral ear). It is employed at an intensity of 50 dBSL, having as reference the mean value of the air conduction tonal thresholds for frequencies 500, 1,000 and 2,000Hz. The child is then instructed to repeat all the words heard, following the order of presentation of these words. An error was considered when a word was omitted, replaced or distorted. We made the quantitative analyses of results for DC and EC situations and the qualitative, that is the auditory effect (AE) and the order effect (OE), the inversion errors (I) and the type A response pattern (type A). As normal values we considered 90% of correct answers for DC/EC, I=1, EA=-4 a +4, EO=-3 a +3 and type A=3 for children of 9 years of age and older; for those with 8 years: DC=80%/EC=75%, I=5, EA=-6 a +4, EO=-4 a +3 and type A=3; for 7 year olds: DC=75%/EC=65%, I=5, EA=-8 a +6, EO=-4 a+10 and type A=6.

Just like the DD test, the SSW test assesses the background-figure skill for verbal sounds^30^ and aims at providing information on the physiological auditory mechanism of verbal sound recognition in dichotic hearing. It also assesses the complex temporal ordering skill, providing information on the physiological auditory mechanism of sound discrimination in sequence, when inversions (I) are present in the SSW.^30^

The children's performance on the tests was classified in: bad performance–when the child did not reach the expected scores for their ages in any of the ears and competitive conditions, and good performance when reached.

Not all the tests proposed and described in the methodology could be applied to the population sampled, because of alterations present in the speech of the sampled population and technical problems.

The data obtained was organized in Tables in order to facilitate analysis and presentation. We carried out a statistical treatment, with a minimum significance level of 5% (p < 0.05).

We used the Mann-Whitney test in order to check for possible differences between the genders, as well as the Wilcoxon Signed Posts Tests in order to check for possible differences between the two sides regarding the variables studied.

## RESULTS

Table 2 presents the descriptive statistics of the special behavioral tests of auditory processing in the population sampled.

**Table 2:** Descriptive statistics of the special behavioral tests for auditory processing in the population sampled.

Tests	Mean	Standard Deviation	Median	Minimum	Maximum
Dichotic tests/Free Field					
MSSNV	2,16	0,90	2,00	0,00	3,00
MSSV	2,42	0,84	3,00	0,00	3,00
LS	4,37	0,76	5,00	3,00	5,00
AFT-R	60,50	40,17	68,30	5,10	146,60
Monotic tests					
SSI/MCI_0OD	65,33	22,32	60,00	30,00	100,00
SSI/MCI_0OE	67,33	25,49	70,00	30,00	100,00
SSI/MCI-10OD	55,33	25,88	50,00	20,00	90,00
SSI/MCI-10OE	57,33	29,15	50,00	20,00	100,00
SSI/MCI-15OD	40,71	21,65	40,00	10,00	80,00
SSI/MCI-15OE	44,29	26,23	40,00	10,00	90,00
Dichotic tests					
SSI/MCC_0OD	99,38	2,50	100,00	90,00	100,00
SSI/MCC-_0OE	98,75	3,42	100,00	90,00	100,00
SSI/MCC-40OD	96,88	10,14	100,00	60,00	100,00
SSI/MCC-40OE	96,25	8,85	100,00	70,00	100,00
DD_OD	73,55	15,64	78,75	42,50	91,25
DD_OE	73,88	17,33	81,25	38,75	95,00
SSW_DC	58,38	29,31	65,00	10,00	92,50
SSW_EC	65,47	18,65	70,00	17,50	82,50
SSW_I	3,24	5,30	1,00	0,00	16,00
SSW_EA	-2,18	6,71	0,00	-16,00	6,00
SSW_EO	-0,76	5,02	-1,00	-12,00	8,00
SSW_TipoA	6,24	3,78	5,00	1,00	14,00

Legend: OD=right ear; OE=left ear; LS=Sound location; MSSV=Verbal sequential memory; MSSNV=non-verbal sequential memory; AFT-R=Revised Auditory Fusion; SSI/MCI=Synthetic sentences with ipsilateral competitive message; SSI/MCC=Synthetic sentences with contralateral competitive message; DD=Digit dichotic; SSW=Alternate disyllables; DC=competitive right; EC=competitive left; I=inversion; EA=auditory effect; EO=order effect.

Only one child was submitted to the PSI test and this child's scores are included with the SSI test data, being called (SSI) on Table 2, Table 3, having in mind its given similar objective and information.

**Table 3:** Distribution of children (percentage) according to the classification of performance in auditory processing behavioral tests.

				Processing Behavioral Tests				
Des.		Diotic			Monotic		Dichotic	
	LS	MSSV	MSSNV	AFT-R	SSIMCI	SSIMCC	DD	SSW
Good	16(84)	12(63)	15(79)	03(5)	03(20)	13(81)	01(5)	01(5)
Poor	03(16)	07(37)	04(21)	14(95)	12(80)	03(19)	18(95)	17(95)
Total	19(100)	19(100)	19(100)	17(100)	15(100)	16(100)	19(100)	18(100)

Legend: Des.= performance

LS=Sound location; MSSV=Verbal sequential memory; MSSNV=Non-verbal sequential memory; AFT-R=Revised Auditory Fusion; SSI/MCI=Synthetic sentences with ipsilateral competitive message; SSI/MCC=Synthetic sentences with contralateral competitive message; DD=Digit dichotic; SSW=Alternate disyllables.

The lack of statistical significance seen by the Mann-Whitney of the variable effect of gender and the Wilcoxon Signed Posts test of the possible differences between both ears, established the non-separation of ears and genders in the presentation of results (Table 3).

The distribution of children according to their performance classification in the diotic, monotic and dichotic tests to which they were submitted is presented on Table 3.

## DISCUSSION

The findings of the present investigation suggest that the children with labio-palatine fissure from the sampled population showed important alterations in their hearing skills assessed by means of their poor performance in the special behavioral tests of auditory processing.

A high percentage of the children in our study (Table 3) showed their worst results in the AFT-R (95%), DD (95%), SSW (95%) and SSIMCI (80%).

Alterations in the AFT-R test, made on the labio-palatine fissure population was also seen by researchers^32^, reporting that of the 30 children assessed in the group with such craniofacial malformation and normal hearing, 22 (74%) had mean auditory fusion thresholds altered, and 19 had positive history of otitis media. In another study^33^, the authors found results from the temporal resolution test suggesting temporal processing alterations in 7 of the 10 children with palatine fissure with a past of otitis media. In contrast to that, scholars^34^ did not find in their studies evidence enough to suggest that otitis media with effusion affects temporal resolution, even after hearing having returned to normal.

We stress the presence of positive history of otitis media as a factor of probable influence on the performance of children in the AFT-R test, because of middle ear alterations in the population with labio-palatine fissure be almost a consensus^35^^,^^36^ thus inferring that it must have been present at some point during their lives, which can impact test results. Literature^35^^,^^36^ shows that children with labio-palatine fissures have longer periods of sensorial privation, caused by middle ear infection when compared to those without this craniofacial malformation.

Having in mind that the AFT-R test is designed to measure temporal resolution–the capacity to detect time intervals between sound stimuli or detect the shortest time within which the individual is able to discriminate between two audible signals^37^^,^^38^, its alteration can result in difficulties to identify small acoustic variations in speech or in interpreting the message heard.^39^

Dichotic tests, such as DD and SSW, are those in which different stimuli are presented to each ear simultaneously^40^ and have been used to explore binaural integration skills. Thus, binaural integration is the skill to process different information presented to both ears, at the same time. A poor performance in binaural integration can be expressed in behavioral symptoms of auditory difficulty in the presence of background noise, as it happens in the difficulty a person has to understand two people talking at the same time^41^^,^^42^. Scholars^43^ found 65% of alterations in the dichotic tests (digits and non-verbal) in children with non-syndromic labio-palatine fissure. In the present investigation, most of the children (95%) had altered results.

Researchers^44^ showed a greater involvement of figure-background skills, binaural integration and sequential memory studying children with history of recurrent otitis in early childhood. They suggested that a fluctuation in hearing, caused by the otitis, can have a negative effect on the individual's own development process–because of poor listening, which can persist despite disease inactivity.

Since binaural integration involves working memory and divided attention^41^, one could consider deficits in these skills influencing the poor performance found in dichotic tests of the present investigation, because even the simplest hearing task is impacted by high level functions not specific to this modality, such as attention, learning, motivation, memory and decision-making processes, and also because attention can facilitate the hearing process.^45^ However, it is important to bear in mind that the type of attention required during a dichotic test is diversified in the literature.^46^

Poor performance in the SSW test happened because of the score reached by the sample population in this test, both for the qualitative and quantitative analyses, which mean values presented on Table 2 are way below what is expected for this age. Alteration in complex temporal ordering skill was seen by the presence of inversions in the SSW test, shown by the descriptive statistical study, reaching values from zero to a maximum of 16, with mean value of 3.24.

Still, regarding SSW qualitative analyses, a variation of −16 to 6 (mean value of −2.18) and of −12 to 8 (mean value of −0.76) was scored in this study, for EA and EO, respectively. Depending on the type (high-low or low-high) of auditory effect (AE) and order effect (OE) observed, it is possible to suggest the presence of alterations in certain areas of the brain, as well as the temporal characteristics stemming from this alteration. Nonetheless, the AE and OE types obtained were not considered in the presentation of this study. A type A standard error was also observed in the population sampled (mean value of 6.24), presenting a maximum value of 14, way above what is expected for this age range. This type of error characterizes difficulties of audio-visual integration.^47^

As to the SSW quantitative analysis, a poor performance (low) in DC and EC in the SSW can suggest dysfunction in the left temporal lobe^48^ and are associated with poor phonemic decoding skills, and it can show poor phonetic skills (affecting reading and spelling), receptive language and articulatory difficulties in the first years^49^. In the present investigation we obtained a mean value of 58.38% and 65.47%, for DC and EC, respectively, below what is expected for this age range.

In regards of the SSI test with ipsilateral competitive message (SSI-MCI), both the mean values and the minimum values are bilaterally way below the expected values for all the signal to noise ratios studied. This results seems to prove that the children of the present study had difficulties performing this monotic task, by means of a selective attention process, once the information presented to a single ear must be separated, using the figure-background skill.^50^ Nonetheless, when the test was carried out with dichotic task of binaural separation (SSIMCC), that is, such a task in which the child has to direct his/her attention to the information presented to one ear, while ignoring the competitive message (story) presented to the other ear, a high percentage (81%) of children with good performance was seen. This skill is important in order to hear and understand speech in noisy enviroments.^51^

The best performances, shown by a high percentage of children (Table 3), happened for three dichotic tests (LS, MSSNV, MSSV) and one diotic (SSI/MCC).

As we analyzed the results from the sound location test (LS), we see that most of the children with labio-palatine fissure (84%) of the sample had adequate mechanism of sound source direction discrimination. This mechanism depends on the process of binaural interaction, that is, with both ears working together^40^^,^^41^^,^^52^ for proper perception of the sound source, having the superior olivary complex and the inferior colliculus as structures responsible for it, both located in the brainstem^40^^,^^53^^,^^54^, as well as the auditory cortex^55^. Alterations in this skill would be justified by an asymmetrical peripheral hearing loss^40^ not present during the exam in the children of our sample.

Having in mind that the performance in the tests that assess sequential memory for non-verbal stimuli (MSSNV) as well as for verbal stimuli (MSSV) have mean values of 2.16 and 2.42 respectively; it allowed us to see that the short term memory skills for the performance of children in these tests proved to be within expected values for this age.

## CONCLUSION

The findings of the present investigation suggest that a high percentage of the children in this study showed their worst results in the Revised Auditory Fusion Dichotic Digits test, SSW, in the SSI-MCI test. The best performances happened for the sound location, sequential memory for non-verbal and verbal sounds and for the synthetic sentences test with contralateral competitive message.
